# Effects of Dietary Energy Levels on Growth Performance, Nutrient Digestibility, Rumen Barrier and Microflora in Sheep

**DOI:** 10.3390/ani14172525

**Published:** 2024-08-30

**Authors:** Xiaolin Wang, Jia Zhou, Mingli Lu, Shoupei Zhao, Weijuan Li, Guobo Quan, Bai Xue

**Affiliations:** 1 Animal Nutrition Institute, Sichuan Agricultural University, Chengdu 611130, China; 2 Chongqing Academy of Animal Sciences, Chongqing 402460, China; 3 Yunnan Animal Science and Veterinary Institute, Kunming 650224, China

**Keywords:** metabolic energy, rumen fermentation, barrier function, microbial community

## Abstract

**Simple Summary:**

Dietary energy level impacts rumen function and the microflora in ruminants, which are closely related to their growth and well-being. This study explored how dietary energy levels affect sheep’s growth performance, nutrient digestibility, rumen fermentation, barrier function, and microflora. Our findings indicated that the average daily weight gain and nutrient digestibility improve with the increase of dietary energy. Meanwhile, the concentration of total volatile fatty acids and propionate in rumen increased, which indicated the enhancement of rumen fermentation. The content and expression of tight junction proteins, which represents rumen barrier function, increased with the increase of dietary energy. In addition, the bacterial diversity in rumen decreased, and the relative abundance of some bacteria like *Prevotellaceae*, *Muribaculaceae*, *Saccharofermentans*, *Prevotella* and *Succiniclasticum* changed at the family and genus levels. Ultimately, the growth performance, fermentation characteristics and barrier function were the best when the daily dietary metabolizable energy was 9.8–10.4 MJ/kg. This study revealed the complex interaction between diet, microbiota, and rumen health, ultimately guiding the development of more effective and scientifically informed animal feeding strategies.

**Abstract:**

Dietary energy is crucial for ruminants’ performance and health. To determine optimal dietary energy levels for growing sheep, we evaluated their growth performance, nutrient digestibility, rumen fermentation, barrier function, and microbiota under varying metabolic energy (ME) diets. Forty-five growing Yunnan semi-fine wool sheep, aged 10 months and weighing 30.8 ± 1.9 kg, were randomly allocated to five treatments, each receiving diets with ME levels of 8.0, 8.6, 9.2, 9.8 or 10.4 MJ/kg. The results showed that with increasing dietary energy, the average daily gain (ADG) as well as the digestibility of dry matter (DM) and organic matter (OM) increased (*p* < 0.05), while the feed conversion ratio (FCR) decreased linearly (*p* = 0.01). The concentration of total VFA (*p* = 0.03) and propionate (*p* = 0.01) in the rumen increased linearly, while rumen pH (*p* < 0.01) and the acetate–propionate ratio (*p* = 0.01) decreased linearly. Meanwhile, the protein contents of Claudin-4, Claudin-7, Occludin and ZO-1 as well as the relative mRNA expression of Claudin-4 and Occludin also increased (*p* < 0.05). In addition, rumen bacterial diversity decreased with the increase of dietary energy, and the relative abundance of some bacteria (like *Saccharofermentans*, *Prevotella* and *Succiniclasticum*) changed. In conclusion, increasing dietary energy levels enhanced growth performance, nutrient digestibility, rumen fermentation, and barrier function, and altered the rumen bacterial community distribution. The optimal dietary ME for these parameters in sheep at this growth stage was between 9.8 and 10.4 MJ/kg.

## 1. Introduction

The rumen, a distinctive digestive organ in ruminants, is intimately linked to their productive performance. A vast array of microorganisms inhabits the rumen, and their stability is a key indicator to evaluate the rumen environment. Malmuthuge et al. reported that certain rumen microorganisms in ruminants facilitated early rumen epithelium development [1], which was the principal interface for microbe-host interactions, resisting pathogen and toxin invasions, and is integral to the rumen’s physical barrier function [2,3]. Lin et al. also indicated that the rumen microflora drove the production of volatile fatty acids (VFAs) to stimulate the growth of rumen epithelia and papillae in lambs and regulated the rumen development [4]. Furthermore, the VFAs produced by fermentation in the rumen have been demonstrated to be involved in the regulation of rumen barrier function by affecting rumen epithelial cell renewal, epithelial integrity and tight junction protein expression [5,6]. Collectively, a stable microbiome and intact rumen epithelium are crucial for sustaining optimal rumen fermentation and barrier function, which not only facilitates nutrient digestion and absorption, but also enhances the production performance and contributes to the overall well-being of the animal [7,8].

The energy in the diet is predominantly utilized to maintain the normal vital activity, basal metabolism and production requirements of the animal, and also determines the feed intake, nutrient digestibility and conversion efficiency [9,10]. There are also several reports that dietary energy affects energy intake and balance as well as body metabolism in dairy cows [11,12]. As the primary substrate for rumen fermentation, diet and its energy levels are also important factors affecting microflora and VFAs production in the rumen. Appropriately elevating the energy density within the diet improves the composition of the rumen microbial community and rumen morphology, thereby enhancing rumen fermentation and the integrity of epithelial tight junctions [13,14]. However, an excessively high energy might induce subacute ruminal acidosis in goats, damaged rumen tissue and destroyed rumen epithelium [15]. Yu et al. also indicated that high dietary energy adversely affected the expression of tight junction proteins like Claudin-1 within the rumen epithelium and the production of VFAs [16].

Currently, research on the effects of dietary energy levels on growth performance and rumen health in sheep has scarcely been reported. Therefore, this comprehensive study aimed to compare the growth performance, nutrient digestibility, rumen fermentation and barrier function in sheep consuming diets with different energy levels, and further evaluated the microbial community structure by 16S rRNA gene sequencing. We hypothesized that dietary energy levels would regulate the rumen bacterial community and affect rumen fermentation and barrier function, and also that there is a certain energy level which is most beneficial for growth performance and nutrient digestion of sheep. Our results may be beneficial for the optimization of dietary energy levels, and ultimately guiding the development of more effective and scientifically informed animal feeding strategies.

## 2. Materials and Methods

The experiment followed animal protection law (Approval number: SCAUAC2019-36) and was performed in accordance with the Experimental Animal Committee of Animal Nutrition Institute, Sichuan Agricultural University.

### 2.1. Animals, Experimental Design and Diet

This study involved 45 healthy, 10-month-old Yunnan semi-fine wool sheep, initially weighing an average of 30.8 ± 1.9 kg, which were randomly assigned to 5 different dietary treatments. The ingredients and chemical composition of these diets are shown in Table 1. The diets were formulated to meet the nutrient requirements for a 30-kg sheep gaining 200 g/day, as per the Nutrient Requirements of Small Ruminants by the National Research Council (NRC, 2007). The metabolic energy (ME) levels of the diets were 8.0, 8.6, 9.2, 9.8, and 10.4 MJ/kg, with the 8.6 MJ/kg diet meeting the target ME requirement. The sheep were housed at the small ruminant research facility of the Animal Nutrition Research Institute at Sichuan Agricultural University, located in Yaan, China, with an altitude of 588 m above sea level. Following the 15-day adaptation period, a 45-day feeding experiment and then a 7-day digestion trial were initiated. Sheep were individually housed in 45 identical pens (2 m × 1.5 m) within a single building, ensuring uniform housing conditions. Throughout the study, sheep had free access to fresh water and their respective experimental diets.

### 2.2. Determination of Growth Performance

The experimental diets were provided ad libitum to the sheep twice a day at 0800 and 1800 h, with meticulous adjustments to the offered amount to ensure a refusal rate of approximately 10%. These diets were offered as a total mixed ration (TMR) to prevent dietary component selection by the sheep. Feed offered and refused were weighed and recorded accurately twice daily, which was then used to calculate the dry matter intake (DMI) for each sheep. The sheep were weighed on days 0 and 45 of the growth measurement period, before their morning feeding. Based on these weights, the average daily gain (ADG) and feed conversion ratio (FCR) were determined.

### 2.3. Determination of Apparent Nutrient Digestibility

A 7-day digestion trial was conducted at the conclusion of the feeding experiment, comprising 2 days for adaptation and the subsequent 5 days for fecal collection. All sheep were moved to individual metabolism cages measuring 1.6 m × 1 m × 1.3 m, with a plastic screen laid on the tray beneath each cage to facilitate fecal collection. Fecal samples from each sheep were collected and quantified daily before feeding throughout the digestion trial’s data collection period. A portion of the collected fresh feces was treated with 5% sulfuric acid (at a concentration of 10%, vol/vol) to prevent the volatilization of ammonia nitrogen. All feed and fecal samples were dried at 55 °C for 48 h, then pulverized, sifted through a 1 mm mesh sieve, and stored in sealed plastic bags at 4 °C for subsequent routine nutrient analysis.

The dry matter (DM) was determined according to AOAC (2000/950.46), ash and organic matter (OM) were obtained according to AOAC (2000/923.03), while crude protein (CP) was determined by the Kjeldahl method (AOAC, 2000/981.10) [17]. The content of neutral detergent fiber (NDF) and acid detergent fiber (ADF) in feed and fecal samples was determined following the previously reported method by Van Soest et al. [18]. Based on the measured nutrient content, apparent digestibility was calculated using the formula: Apparent digestibility = [(% of nutrient intake − % of nutrient in feces)/% of nutrient intake] × 100%.

### 2.4. Collection of Rumen Sample

At the conclusion of the trial, six sheep from each treatment, representative of the average weight, were slaughtered, totaling 30. After the final feeding, they were killed through jugular exsanguination 3 h later, and then the rumen fluid and epithelial tissue were collected and cryopreserved. The rumen fluid was extracted using a medicine spoon after exposing the rumen, and was filtered through 4 layers of cheesecloth, and the filtrate was divided into two fractions. In one fraction, 5 mL of filtrate was combined with 1 mL of 25% metaphosphoric acid, mixed thoroughly, and centrifuged at 3000× *g* for 15 min at 4 °C. The supernatant was then isolated and stored at −20 °C for analysis of rumen fermentation parameters. The remaining 3 mL of filtrate was transferred to a cryopreservation tube and preserved in liquid nitrogen for microbial community analysis. Additionally, the ventral sac of the rumen beneath the reticulum was removed using surgical scissors, rinsed gently with PBS buffer, and the epithelial tissue was placed in a 2 mL cryopreservation tube, wrapped in aluminum foil, and stored at −80 °C for tight junction analysis.

### 2.5. Determination of Rumen Fermentation

The rumen pH was promptly measured with a portable pH meter (PB-10; Sartorius Co., Göttingen, Germany) following the extraction of rumen fluid. The ammonia nitrogen concentration was assessed using the phenol/hypochlorite method, as described by Broderick and Kang [19]. Volatile fatty acid (VFA) concentrations, including total VFAs, acetate, propionate, and butyrate, were analyzed using gas chromatography (CC-8A; Shimadzu Corp., Kyoto, Japan), according to the method of Stewart and Duncan [20].

### 2.6. Determination of Rumen Epithelial Tight Junction

Concentrations of tight junction proteins in rumen epithelium, including Claudin-1, Claudin-4, Claudin-7, occludin, and zonula occludens-1, were quantified with ELISA kits provided by Jiangsu Enzyme Immunity Experimental Co., Ltd. (Jiangsu, China). Furthermore, the mRNA expression of tight junction proteins in the rumen epithelium were quantified. Total RNA from rumen epithelium was extracted with TRIzol reagent (Invitrogen, Carlsbad, CA, USA) following the manufacturer’s protocol, and RNA concentration and purity were assessed using a NanoDrop 2000 spectrophotometer (Thermo Scientific, Waltham, MA, USA). Subsequently, the total RNA was reverse-transcribed with the RevertAid First Strand cDNA Synthesis Kit (Thermo Scientific, Waltham, MA, USA). Real-time PCR was conducted using the Platinum SYBR Green qPCR SuperMix-UDG with ROX kit (Catalog No. 11748-100, Thermo, USA), with primer sequences detailed in Table 2. PCR conditions included an initial denaturation step at 95 °C for 2 min, followed by 40 cycles of amplification (95 °C for 15 s, 60 °C for 30 s, and 72 °C for 10 s). GAPDH served as an endogenous control for the relative quantification of gene expression, which was calculated with the 2^−ΔΔCt^ method.

### 2.7. Determination of Rumen Microbial Community

The bacterial community in the rumen fluid was analyzed by high-throughput sequencing of the 16S rRNA gene at Biomarker Technologies Co., Ltd. (Beijing, China). Bacterial genomic DNA was extracted from rumen fluid samples using a DNA extraction kit [21] according to the manufacturer’s instructions, and the concentration and purity of the DNA were monitored by 1% agarose gel electrophoresis and NanoDrop2000 (Thermo Fisher Scientific, Wilmington, NC, USA). The DNA sample was aliquoted into a sterile, enzyme-free centrifuge tube and diluted to a final concentration of 1 ng/μL with sterile water. The diluted DNA served as a template for PCR amplification, conducted with Phusion^®^ High-Fidelity DNA Polymerase (M0530S, New England Biolabs, Arlington, VA, USA) and specific primer with the barcode for ensuring both efficiency and precision in the amplification process with primer sequence 341F (5′-CCTAYGGGRBGCASCAG-3′) and 806R (5′-GGACTACNNGGGTATCTAAT-3′). The total reaction system was 30 μL, containing 15 μL of Phusion Master Mix (2X), 3 μL of Primer (2 μM), 10 μL of genomic DNA (1 ng/μL) and 2 μL of sterile ultrapure water. The PCR products were mixed thoroughly according to their concentration. Subsequently, the PCR products were resolved by 2% agarose gel electrophoresis and purified using the GeneJET PCR Purification Kit (Thermo Scientific, USA). The purified PCR products were used for constructing a sequenced genomic library with the Ion Plus Fragment Library Kit 48 rxns (Thermo Scientific, USA), followed by Qubit quantification, library validation, and sequencing on the Ion S5TMXL system (Thermo Scientific, USA). The raw read count for the rumen fluid samples was 2,466,728, as determined by Cutadapt V1.9.1 software [22]. Then, the chimera sequences were removed using Trimmomatic software version 0.33 [23] and 2,327,826 clean reads were obtained for the rumen fluid samples. Clean reads were clustered into operational taxonomic units (OTUs) at a 97% sequence similarity threshold using Uparse software version 7.0.1001 [24]. Representative sequences for each OTU were aligned to the Silva database, yielding a classification of the bacterial community at the phylum, class, order, family, and genus levels (only family and genus levels were shown). The clustering analysis revealed 17 phyla, 26 classes, 40 orders, 61 families, 139 genera, and 155 species of bacteria in the ruminal samples. Alpha diversity indices, including Chao1, Ace, Shannon, and Simpson, as well as the relative abundances of bacteria in the rumen fluid samples, were calculated using QIIME software version 1.9.1 [25].

### 2.8. Statistical Analysis

The effect size was calculated and the power was analyed before the experiment: statistical power analysis was performed with α = 0.05 and power = 0.80 using Sample Power Version 1.0 (IBM SPSS, Chicago, IL, USA), and with 6 sheep per treatment group, a 10% difference between treatment means for most variables would be found with a power of 80% or greater, which supports the adequacy of the sample size. Data regarding growth performance, nutrient digestibility, rumen fermentation, tight junctions, and bacterial diversity in sheep were analyzed using ANOVA via the GLM procedure in SPSS software version 22.0 (IBM SPSS, Chicago, IL, USA). Subsequently, linear and quadratic regression models were applied to assess linear and nonlinear relationships. All results were reported as least squares means. The Tukey multiple comparison test was used to determine statistical significance at *p* ≤ 0.05.

## 3. Results

### 3.1. Growth Performance

The growth performance of sheep with different treatments is summarized in Table 3. With the increase of dietary energy, the ADG increased linearly (*p* < 0.01), and was higher in ME 9.8 and ME 10.4 than in ME 8.0 and ME 8.6 (*p* = 0.05); the DMI increased linearly (*p* = 0.04) and quadratically (*p* < 0.01), and was higher in ME 9.2 than in ME 8.0 and ME 10.4 (*p* < 0.01). Ultimately, the FCR decreased in a linear manner (*p* < 0.01), and was lower in ME 9.8 and ME 10.4 than in ME 8.0 (*p* = 0.04). The initial body weight and final body weight almost remained unchanged (*p* > 0.05) under all the treatments tested. 

### 3.2. Apparent Nutrient Digestibility

Table 4 displays the apparent nutrient digestibility in sheep fed diets varying in energy content. The digestibility of DM and OM increased in a linear and quadratic manner with the elevated dietary energy (*p* < 0.05), and were higher in ME 9.8 and ME 10.4 than in ME 8.0 (*p* = 0.04, *p* = 0.02, respectively). In addition, the digestibility of CP, NDF and ADF did not differ (*p* > 0.05) among treatments.

### 3.3. Rumen Fermentation

The pH value as well as the concentration of ammonia nitrogen and VFAs in the rumen of sheep with different treatment are illustrated in Table 5. With the increase of dietary energy, the pH value linearly decreased (*p* < 0.01) and was lower in ME 9.8 and ME 10.4 than in ME 8.0 (*p* < 0.01), while the concentration of total VFAs linearly increased (*p* = 0.03) and was higher in ME 10.4 than in ME 8.0 (*p* = 0.05). In addition, the propionate concentration (*p* = 0.05) in ME 10.4 increased while the acetate–propionate ratio (*p* = 0.03) decreased compared with ME 8.6. The concentration of ammonia nitrogen, acetate and butyrate were almost constant (*p* > 0.05) in all treatments.

### 3.4. Rumen Epithelial Tight Junction

The content and relative mRNA expression of tight junction proteins in the rumen of sheep consuming diets with different energy levels are shown in Table 6. On the one hand, with the increase of dietary energy, the protein content of Claudin-4 and Claudin-7 increased in a linear manner (*p* < 0.01), and were higher in ME 9.8 and ME 10.4 than in ME 8.0 (*p* = 0.01, *p* = 0.02, respectively); the protein content of Occluding and ZO-1 ascended linearly and quadratically (*p* < 0.05), and were higher in ME 9.8 than in ME 8.0 (*p* < 0.05, *p* = 0.02, respectively). On the other hand, the relative mRNA expression of Claudin-4 and Occludin linearly increased (*p* = 0.01, *p* < 0.01, respectively), and were higher in ME 10.4 than in ME 8.0 and ME 8.6 (*p* < 0.05).

### 3.5. Rumen Microbial Community

**Figure 1:**
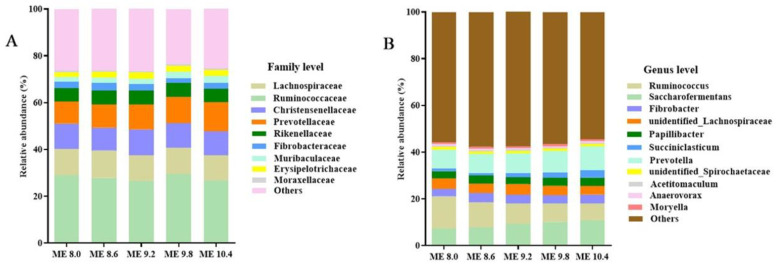
Effects of dietary energy levels on the relative abundances (>0.5%) of bacterial family and genus in the ruminal fluids of sheep. (**A**) The bacterial composition at family level. (**B**) The bacterial composition at genus level.

The alpha diversity in the rumen of sheep fed different diets is presented in Table 7. The Simpson index elevated linearly (*p* = 0.03) with the increase of dietary energy and was higher in ME 9.8 and ME 10.4 than ME 8.0 (*p* = 0.04). However, dietary energy had no influence on the effects of Chao1, Shannon and ACE (*p* > 0.05). The bacterial community at the family and genus level in the rumen fluids among the different treatments were illustrated in Figure 1. At the family level, the relative abundance of *Prevotellaceae* (ME 8.0 vs. ME 10.4 = 9.39% vs. 12.4%) and *Muribaculaceae* (ME 8.0 vs. ME 10.4 = 2.13% vs. 3.06%) increased with the increase of dietary energy. At the genus level, with the increase of dietary energy, the relative abundance of *Saccharofermentans* (7.36% and 10.8% in ME 8.0 and ME 10.4, respectively), *Prevotella* (7.95% and 10.2% in ME 8.0 and ME 10.4, respectively) and *Succiniclasticum* (1.29% and 3.08% in ME 8.0 and ME 10.4, respectively) increased, but the relative abundance of *Ruminococcus* decreased.

## 4. Discussion

The growth performance of animals is closely linked to their DMI, heavily influenced by the nutritional composition of their diet, particularly its energy content. Dietary energy regulates animal feeding behavior by influencing hunger [26]. Fang et al. [27] reported that an increase in dietary energy was usually accompanied by a decrease in the daily feed intake. However, Tovar-Luna et al. [28] observed that feeding a low-energy diet was similar to feeding a high-energy diet: both reduced the DMI of goats. Our study also indicated that the DMI in sheep increased when ME level increased from 8.0 to 9.2 MJ/kg but decreased when ME level continued to rise to 10.4 MJ/kg. Ensuring appropriate energy levels offered to animals is essential for maximizing the growth efficiency. Wang et al. [29] reported that the ADG of Hu sheep increased and the FCR decreased when the dietary ME level increased from 9.17 to 10.41 MJ/kg. However, Ríos-Rincón et al. [30] compared the growth performance of fattening lambs fed two diets with higher energy levels, and found no difference in ADG, which might be due to the fact that the positive regulation of dietary energy on weight gain performance was more easily observed at lower ME levels and limited when energy density exceeded a certain threshold. Similarly, in our study, sheep treated with ME 9.8 and ME 10.4 had higher ADG and lower FCR compared with ME 8.0, but there was no difference between them, indicating that the dietary ME of 9.8–10.4 MJ/kg was conducive to the growth performance of sheep at this growth stage.

The variation in nutrient digestibility among sheep is dependent on the chemical composition of their diets. The dietary ME provided to sheep exhibits variation, with a corresponding fluctuation in the levels of NDF and ADF, as calculated from Table 1. The dietary energy levels influence ruminal and intestinal fermentation patterns and regulate the microflora, thereby affecting the nutrient utilization efficiency of animals [31]. However, the contact of digestive enzymes with the substrate might be limited, thus inhibiting the digestion and utilization of nutrients when the fiber content of the diet was high [32,33]. Several reports stated that feeding diets with higher energy and lower fiber content improved the apparent and true nutrient digestibility, including DM and starch in dairy cows [34,35]. In our study, the digestibility of DM and OM were higher in ME 9.8 and ME 10.4, which was likely due to the higher energy and lower fiber component of their diets.

A high fermentable carbohydrate diet provides more fermentation substrate to the rumen, increasing the fermentation rate of the rumen to such an extent that it exceeds the rate of absorption and buffering, thereby promoting the production of VFAs and leading to a decrease of pH value in the rumen [36]. Ruminal fermentation pattern is primarily determined by the microbial communities participating in the fermentation process and the substrate type [37]. Carbohydrates readily degradable by rumen bacteria are conducive to propionate production [38]. Olijhoek et al. [31] observed that feeding high-energy diets changed rumen VFA distribution and increased the molar proportion of propionate in dairy cows. Zhou et al. [39] reported that the ruminal fermentation pattern of sheep changed from acetate to propionate, butyrate and valerate with the increase of dietary energy, and finally the molar proportion of propionate and butyrate increased. In our study, when the dietary ME level increased from 8.6 to 10.4 MJ/kg, the rumen propionate concentration in sheep increased (10.7 vs. 15.3 mM), while the acetate–propionate ratio decreased (3.41 vs. 2.23), suggesting that rumen fermentation performance was improved. Cui et al. [40] indicated that higher concentrations of propionate increased energy conversion efficiency, which was crucial for growth and development of animals. In addition to the impact of the fermentation substrate type, the increase in the abundance of rumen bacteria producing propionate like *Prevotella* and *Succiniclasticum* [41] under high-energy treatment could not be ignored. Rumen pH was related to total VFA production and also regulated by dietary energy. Liu et al. [9] observed that the rumen total VFA concentration of yaks increased with the increase of dietary energy, while the pH value decreased. Ahmad et al. [42] further reported that the effect of high energy diet on rumen VFA production and pH value might be achieved by increasing the mRNA abundance of VFA transporter genes like MCT1, DRA and PAT1. In our study, the rumen total VFA concentration and pH value of sheep showed similar changes with the increase of dietary energy, indicating enhanced rumen fermentation. However, Villot et al. [43] reported that continuous feeding of high energy diets increased the risk of subacute rumen acidosis (SARA). Although He et al. [44] induced SARA in sheep by feeding diets with ME of 10.05 MJ/kg, and the ME 10.4 treatment in our study exceeded this energy level, rumen pH in sheep under this treatment still remained within the normal range and no SARA occurred. A possible explanation is that the proportion of concentrate in each diet did not change in this study. Altogether, the diet with ME level 10.4 MJ/kg was more beneficial to rumen fermentation of sheep in our study.

The rumen barrier helps to decrease the invasion of pathogenic microorganisms, sustaining rumen homeostasis and overall health, a process that is intimately linked to the tight junction proteins of the rumen epithelium. Hu et al. [45] observed that in subacute ruminal acidosis, the expression of rumen epithelial tight junction protein was down-regulated, leading to structural damage and dysfunction of the epithelial barrier. The response of the rumen epithelium, including tight junctions, gap junctions and inflammatory responses of the epithelium, to dietary energy was probably related to the appropriate concentration of VFA and pH in the rumen [46]. Hu et al. [47] reported that energy restriction in the diet reduced the concentration of acetate, propionate, butyrate and total VFA, while it raised rumen pH, which impaired rumen epithelial barrier function of sheep. Świerk et al. [48] also demonstrated that increasing dietary energy concentration had a positive effect on the mRNA expression of rumen epithelial tight junction proteins like Claudin-1, Claudin-4, Claudin-7 and Occluding in rams. In our study, the tight junction protein contents and mRNA expression increased with the increase of dietary energy, which suggested that the permeability decreased and physical barrier was enhanced. Rumen butyrate has been reported as a signaling molecule to improve the tight junctions of the rumen epithelium [49], but in this study, the reason for improvement of the rumen barrier by increasing dietary energy was obviously not that increase, but probably the rumen pH. The rumen pH of sheep fed different energy diets was not lower than 5.80, indicating that their rumen function was not challenged by acidic conditions [50], in which case improving the dietary energy and thereby reducing rumen pH by increasing the intake of fast-degrading carbohydrates might stimulate rumen epithelial function to enhance the rumen barrier, rather than challenge it [48]. In addition, an increase in rumen VFA concentration and a decrease in pH could favor peroxisome formation, which is important for maintaining the integrity of epithelial cells [46]. However, although rumen epithelial cells could tolerate a low pH (5.10) environment for several hours, continued exposure still damaged tight junctions and disrupted barrier function [51]. In some studies, excessively high dietary energy intake decreased rumen pH, and might weaken or even damage the rumen epithelial barrier [52]. Li et al. [53] reported that increasing soluble carbohydrates in the diet enhanced tight junction protein-like Occluding expression in lambs’ rumen, but a sustained increase in dietary energy activated NF-κB and MAPK pathways in the rumen epithelium, leading to rumen barrier damage. Shen et al. [46] compared the rumen epithelial function of goats at three dietary ME levels, and found that the rumen barrier was improved with ME of 11.4 MJ/kg compared with a diet with ME of 10.1 MJ/kg, while the rumen barrier was damaged when ME level continued to rise to 14.0 MJ/kg. In our study, the concentration and expression levels of tight junction proteins were higher when dietary ME was 9.8–10.4 MJ/kg, indicating the best barrier function. However, with the continuous increase of dietary energy, we cannot determine whether the barrier function will decline, which needs to be further investigated in future studies.

The rumen microbial community affects the growth performance and rumen health of ruminants by participating in the digestion process and regulating rumen epithelium [3]. The coverage values in ruminal fluid samples of sheep treated with different treatments exceeded 0.98, suggesting that the rumen microbiota was comprehensively represented, and the sequencing results accurately mirrored the true composition of bacteria within the rumen. According to the results of alpha diversity, the rumen bacteria richness did not change, while the rumen bacterial diversity decreased with the increase of dietary energy in our study. Liu et al. [54] found that increasing the dietary concentrate proportion to raise energy levels reduced rumen bacterial diversity in sheep. Wu et al. [55] indicated that the low pH environment in the rumen with a large amount of VFA production could not be conducive to the survival of some poorly tolerated rumen bacteria, which might be the reason for the lower bacterial diversity of ME 9.8 and ME 10.4 in our study. Additionally, a reasonable explanation was that the increase of dietary energy increased the relative abundance of dominant bacteria like *Prevotella* in the rumen, which can inhibit the metabolic activities of a relatively low number of bacteria, thereby reducing bacterial diversity.

Dietary energy levels consistently affect the relative composition of the rumen bacterial community in ruminants [16,42]. Bacteria from the *Ruminococcaceae*, *Lachnospiraceae*, *Christensenellaceae* and *Prevotellaceae* families seem to dominate the core microflora in the rumen in our study, regardless of dietary energy level. The relative abundance of *Prevotellaceae* and *Muribaculaceae* in the rumen improved with the increase of dietary energy, suggesting that the capability for carbohydrate metabolism of sheep was enhanced [56,57]. The *Muribaculaceae* family has also been shown to be involved in the formation of mucus layers and the maintenance of barrier function [58]. Feeding a high-energy diet helped to increase the number of *Prevotella* in the rumen [59], which is also confirmed in our study. Wang et al. [60] reported that elevated levels of *Prevotella* in the rumen enhance the expression of succinate-CoA synthetase, which indirectly supports the production of propionate. The relative abundance of *Saccharofermentans* and *Succiniclasticum* in the rumen elevated with the increase of dietary energy, which promoted the degradation of polysaccharides to produce VFA-like propionate [41,61]. In addition, the competitive interaction among dominant bacterial genus was likely the primary driver behind the observed decline in the relative abundance of *Ruminococcus*. Dietary energy probably exerts additional effects on the genes and metabolites of enzymes involved in energy metabolism of sheep by regulation of microbial communities [62], but this needs to be further investigated in future studies.

## 5. Conclusions

Elevating the ME levels in the diet enhanced growth performance, nutrient digestibility, and total VFA concentration in the rumen, improved rumen fermentation and barrier function, and altered the rumen bacterial community structure. Considering all factors in our study, the optimal dietary ME for sheep in this growth phase is 9.8 to 10.4 MJ/kg. This study highlights the importance of maintaining appropriate dietary energy levels to improve ruminant performance and health, and provides empirical evidence for developing nutritional strategies specific to sheep.

## Figures and Tables

**Table 1:** Ingredients and chemical composition of diets offered to sheep.

Items	Treatments				
	ME 8.0	ME 8.6	ME 9.2	ME 9.8	ME 10.4
Ingredient composition, % of DM					
Corn	11.0	19.4	28.2	34.2	35.0
Wheat bran	31.2	21.8	12.00	5.00	0.00
Soybean meal	6.00	7.00	8.00	9.00	9.00
Corn starch	0.00	0.00	0.00	0.00	4.20
Sodium chloride	0.50	0.50	0.50	0.50	0.50
Sodium bicarbonate	0.30	0.30	0.30	0.30	0.30
Premix ^1^	1.00	1.00	1.00	1.00	1.00
Corn Silage	10.0	21.0	33.0	40.0	50.0
Wheat Straw	40.0	29.0	17.0	10.0	0.0
Chemical composition, % of DM					
ME ^2^, MJ/kg	8.00	8.60	9.20	9.80	10.4
CP	10.4	10.3	10.5	10.7	10.5
NDF	48.3	46.7	44.1	43.2	41.5
ADF	22.0	20.9	19.6	19.6	19.2
Calcium	0.66	0.63	0.65	0.62	0.64
Phosphorus	0.33	0.34	0.33	0.35	0.32

^1^ The premix provided the following, per kilogram: nicotinic acid 1.2 g, Cu 2 g, Fe 10 g, Zn 6 g, Mn 5 g, I 100 mg, Co 55 mg, Se 35 mg, vitamin A 500,000 IU, vitamin D3 200,000 IU, vitamin E 850 IU. ^2^ The ME was calculated and the CP, NDF, ADF, Calcium and Phosphorus were measured.

**Table 2:** Sequences of oligonucleotide primers for real-time quantitative fluorescence PCR.

Genes	Accession No.	Primes (5′-3′)
Claudin-1	NM_001185016.1	F: GCTGCTGCTTCTCTCTGCCTTC
		R: GCCTGGGTGTTGGGTAAGATGTTG
Claudin-4	NM_001185017.2	F: TCATCGGCAGCAACATCGTCAC
		R: CAGCAGCGAGTCGTACACCTTG
Claudin-7	NM_001185018.1	F: GCAGAGCACCGGCATGATGAG
		R: CAGCACCAGGGAAACCACCATTAG
Occludin	XM_015101255.2	F: GCCTGTGTTGCCTCCACTCTTG
		R: CATAGCCATAGCCACTTCCGTAGC
ZO-1 ^1^	XM_015101953.2	F: ACCATCACGCCAGCATACAATCG
		R: GCTTTGGAGGACAGGTCAGGTTTG
GAPDH	NM_001190390.1	F: CGGCACAGTCAAGGCAGAGAAC
		R: CACGTACTCAGCACCAGCATCAC

^1^ ZO-1 = zonula occludens-1.

**Table 3:** Effects of dietary energy levels on growth performance in sheep.

Items	Treatments					SEM	*p*-Value	*p* _Linear_	*p* _Quadratic_
	ME 8.0	ME 8.6	ME 9.2	ME 9.8	ME 10.4				
Initial BW, kg	30.2	31.5	30.3	30.8	31.2	0.92	0.60	0.75	0.87
Final BW, kg	33.9	36.3	35.8	37.0	37.6	1.91	0.37	0.06	0.72
ADG, g/d	81.5 ^c^	99.3 ^bc^	122.0 ^ab^	138.3 ^a^	142.9 ^a^	22.6	0.05	<0.01	0.58
DMI, g/d	889.6 ^c^	1031.6 ^ab^	1081.0 ^a^	1036.8 ^ab^	983.5 ^b^	21.9	<0.01	0.04	<0.01
FCR	12.0 ^a^	10.4 ^ab^	8.15 ^ab^	7.21 ^b^	6.54 ^b^	1.13	0.04	0.01	0.48

Mean values with different superscripts within the same row were significantly different (*p* ≤ 0.05), while mean values with no letter superscripts within the same row mean no significant difference (*p* > 0.05).

**Table 4:** Effects of dietary energy levels on apparent nutrient digestibility in sheep.

Items	Treatments					SEM	*p*-Value	*p* _Linear_	*p* _Quadratic_
	ME 8.0	ME 8.6	ME 9.2	ME 9.8	ME 10.4				
DM	63.1 ^b^	65.8 ^ab^	67.1 ^ab^	69.9 ^a^	70.5 ^a^	0.92	0.04	0.02	0.01
OM	65.6 ^b^	67.9 ^ab^	70.1 ^ab^	71.2 ^a^	71.8 ^a^	0.78	0.02	0.01	0.02
CP	65.5	63.7	66.5	65.4	65.9	0.85	0.36	0.66	0.91
NDF	56.6	55.8	56.8	54.6	55.7	1.89	0.52	0.38	0.67
ADF	48.3	47.4	48.7	48.4	48.2	1.38	0.61	0.45	0.69

Mean values with different superscripts within the same row were significantly different (*p* ≤ 0.05), while mean values with no letter superscripts within the same row mean no significant difference (*p* > 0.05).

**Table 5:** Effects of dietary energy levels on rumen fermentation in sheep.

Items	Treatments					SEM	*p*-Value	*p* _Linear_	*p* _Quadratic_
	ME 8.0	ME 8.6	ME 9.2	ME 9.8	ME 10.4				
pH	7.06 ^a^	6.97 ^ab^	6.94 ^ab^	6.85 ^bc^	6.79 ^c^	0.05	<0.01	<0.01	0.92
Ammonia-N, mg/dL	11.1	10.7	12.1	11.3	11.8	0.51	0.08	0.09	0.77
Total VFA, mM	50.7 ^b^	53.2 ^ab^	52.2 ^ab^	54.0 ^ab^	55.3 ^a^	1.35	0.05	0.03	0.09
Acetate, mM	34.4	35.3	32.6	34.4	33.8	1.01	0.14	0.40	0.61
Propionate, mM	11.6 ^ab^	10.7 ^b^	13.7 ^ab^	12.8 ^ab^	15.3 ^a^	0.74	0.05	0.01	0.51
Butyrate, mM	4.76	7.27	5.80	6.83	6.20	0.79	0.10	0.28	0.15
Acetate–propionate	3.13 ^ab^	3.41 ^a^	2.46 ^ab^	2.88 ^ab^	2.23 ^b^	0.26	0.03	0.01	0.65

Mean values with different superscripts within the same row were significantly different (*p* ≤ 0.05), while mean values with no letter superscripts within the same row mean no significant difference (*p* > 0.05).

**Table 6:** Effects of dietary energy levels on rumen epithelial tight junction in sheep.

Items	Treatments					SEM	*p*-Value	*p* _Linear_	*p* _Quadratic_
	ME 8.0	ME 8.6	ME 9.2	ME 9.8	ME 10.4				
Protein contents									
Claudin-1	32.9	43.5	43.5	39.9	41.4	1.69	0.17	0.26	0.13
Claudin-4	9.87 ^c^	13.4 ^bc^	15.2 ^ab^	16.4 ^ab^	17.6 ^a^	0.54	0.01	<0.01	0.24
Claudin-7	4.53 ^b^	5.44 ^ab^	5.99 ^a^	5.97 ^a^	6.18 ^a^	0.13	0.02	<0.01	0.08
Occludin	21.1 ^b^	24.3 ^ab^	25.6 ^ab^	28.5 ^a^	25.8 ^ab^	0.59	<0.01	<0.01	0.01
ZO-1	14.3 ^b^	17.4 ^ab^	18.4 ^ab^	21.1 ^a^	18.6 ^ab^	0.44	0.02	<0.01	<0.01
Relative mRNA expression									
Claudin-1	1.26	1.34	1.27	1.31	1.19	0.13	0.83	0.60	0.39
Claudin-4	1.29 ^b^	1.32 ^b^	1.47 ^ab^	1.65 ^a^	1.72 ^a^	0.10	<0.01	0.01	0.70
Claudin-7	1.22	1.18	1.31	1.37	1.26	0.10	0.35	0.22	0.41
Occludin	1.08 ^b^	0.99 ^b^	1.16 ^ab^	1.24 ^ab^	1.38 ^a^	0.09	0.01	<0.01	0.18
ZO-1	1.14	1.20	1.18	1.27	1.32	0.12	0.28	0.37	0.26

Mean values with different superscripts within the same row were significantly different (*p* ≤ 0.05), while mean values with no letter superscripts within the same row mean no significant difference (*p* > 0.05).

**Table 7:** Effects of dietary energy levels on rumen microbial diversity in sheep.

Items	Treatments					SEM	*p*-Value	*p* _Linear_	*p* _Quadratic_
	ME 8.0	ME 8.6	ME 9.2	ME 9.8	ME 10.4				
Coverage	0.98	0.98	0.98	0.98	0.98	<0.01	0.45	0.37	0.62
Chao1	855.7	952.5	853.9	845.3	840.2	23.0	0.75	0.43	0.60
Shannon	7.19	7.26	7.30	7.75	7.36	0.07	0.21	0.08	0.34
Simpson	0.97 ^b^	0.98 ^ab^	0.98 ^ab^	0.99 ^a^	0.99 ^a^	<0.01	0.04	0.03	0.51
ACE	853.8	948.4	853.6	844.9	839.9	23.2	0.33	0.46	0.59

Mean values with different superscripts within the same row were significantly different (*p* ≤ 0.05), while mean values with no letter superscripts within the same row mean no significant difference (*p* > 0.05).

## Data Availability

All data presented in this article are available upon request to the author if necessary.
